# Association of American Medical Editors

**Published:** 1875-05-15

**Authors:** 


### ASSOCIATION OF AMERICAN MEDICAL EDITORS.

THE seventh anniversary meeting of this organization was held in the parlor of the Galt House, Louisville, Ky., on the evening of May 3d, 1875. There were present Dr. W. S. Edgar, of the *St. Louis Medical and Surgical Journal;* Dr. W. K. Bowling, of the *Nashville Journal of Medicine;* Drs. L. P. and D. W. Yandell, and T. Parvin, of the *American Practitioner;* Dr. A. N. Bell, of the *Sanitarian;* Dr. H. C. Wood, of the *Philadelphia Medical Times;* Dr. N. S. Davis, of the MEDICAL EXAMINER; Dr. Culbertson, of Cincinnati, and Dr. Bixbey, of Boston.

The meeting was called to order by the President, Dr. W. S. Edgar, of St. Louis, who read an interesting address, criticizing the present system of medical education, advocating the adoption of measures to insure a better preliminary education on the part of young men before entering upon the study of medicine, and the establishment of boards in each State to examine and license men to practice, independent of the schools or of the college diploma. The subjects embraced in the address were discussed by nearly all the members present. The following officers were elected for the ensuing year:

President, Dr. A. N. Bell, of Brooklyn, N. Y.; Vice-President, Dr. H. C. Wood, of Philadelphia; Secretary, Dr. F. H. Davis, of Chicago.

The Association adjourned to meet on Monday evening preceding the first Tuesday in June, 1876, in Philadelphia.

